# Raised circulating corticosterone inhibits neuronal differentiation of progenitor cells in the adult hippocampus

**DOI:** 10.1016/j.neuroscience.2005.08.073

**Published:** 2006

**Authors:** E.Y.H. Wong, J. Herbert

**Affiliations:** a Department of Anatomy, University of Cambridge, Downing Street, Cambridge CB2 3DY, UK; b Cambridge Centre for Brain Repair, University of Cambridge, Robinson Way, Cambridge CB2 2PY, UK

**Keywords:** neurogenesis, differentiation, dentate gyrus, hippocampus, cortico steroids, progenitor cells, ADX, adrenalectomy/adrenalectomized, DCX, doublecortin

## Abstract

Neurons are added throughout life to the dentate gyrus of the hippocampus of the mammalian brain. Progenitors residing in the dentate gyrus progress through three distinct stages of adult neurogenesis: proliferation, survival and differentiation. One of the most potent factors which regulates adult neurogenesis is adrenal-derived glucocorticoids. Raised levels of glucocorticoids suppress progenitor division, while removal of glucocorticoids by adrenalectomy stimulates proliferation of these cells in the dentate gyrus. We have recently reported that both pre- and post-mitotic corticoid environments powerfully regulate survival of progenitor cells in a time-dependent manner. However, it is unknown if glucocorticoids alter the process of neuronal differentiation, since not all of the newly-formed cells acquire a neuronal fate during development. Here we employ triple immuno-fluorescence staining techniques to phenotype surviving progenitor cells 28 days after labeling. Results show that high levels of corticosterone (the major glucocorticoid in rodents) either before or after progenitor labeling discouraged the acquisition of neuronal fate. Similar to its effect on survival, post-mitotic corticosterone also regulates neuronal differentiation in a time-dependent fashion, but this action is most prominent from around 19–27 days after the cells were born. In contrast, a corticoid-free environment either before or after progenitor proliferation did not affect neuronal differentiation. Combining these data with previous survival data obtained from the same animals allowed us to estimate the total number of neurons formed resulting from different corticoid treatments. Raised corticosterone significantly reduced neuronal production while adrenalectomy resulted in significantly higher number of neurons in the adult male rat hippocampus.

Neurogenesis in the adult brain is now well established since its first report 40 years ago (Altman and Das, 1966). Two regions, the subventricular zone and the dentate gyrus of the hippocampus, are known to produce new neurons throughout life (Cameron et al 1993, Corotto et al 1993). Neuron formation during adulthood occurs in diverse species including rodents (Cameron et al., 1993), monkeys (Gould et al., 1999b) and humans (Eriksson et al., 1998). The hippocampus is a structure involved in memory formation (Izquierdo and Medina, 1997), consolidation (Nadel and Moscovitch, 1997) and retrieval (de Quervain et al., 2000), and such functions have raised the possibility that constitutively produced new neurons may play crucial roles in learning and memory (Kempermann, 2002). Since drugs known to be effective antidepressants stimulate neurogenesis (Santarelli et al., 2003), there is recent clinical interest in the possibility that either the onset of depression or its treatment may be associated with changes in neurogenesis in the dentate gyrus (Sapolsky 2004, Malberg and Schechter 2005).

A wide range of factors has been shown to regulate adult neurogenesis. Learning (Gould et al., 1999a), experience (Kempermann et al., 1997), neural injuries (Liu et al 1998, Haughey et al 2002) and growth factors (Kuhn et al 1997, Aberg et al 2000) stimulate neurogenesis while ageing (Kuhn et al., 1996), psychological/physical stress (Gould et al 1997, Pham et al 2003), radiation (Peissner et al., 1999), alcohol (Nixon and Crews, 2002) and many others suppress this process.

The fate of progenitors in the dentate gyrus can be classified into three distinct stages: proliferation, survival and differentiation. Progenitor cells divide and give rise to daughter cells which form clusters at the subgranular zone of the dentate gyrus. A proportion of these newly-formed cells undergo apoptosis and only about half of them survive (Wong and Herbert, 2004). The surviving progenitors then differentiate into neurons, glia and other cell types (Monje et al., 2002). Modulators of neurogenesis may affect a particular developmental stage (Kempermann et al., 1997), and therefore it is important to study the three stages separately in order to understand how the production of neurons is regulated in the adult brain.

Glucocorticoids (cortisol in humans, or corticosterone in rodents) are adrenal-derived steroids. Glucocorticoids enter the brain through the blood–brain barrier, and their levels are heightened during stressful events. Raised levels of glucocorticoids suppress proliferation of progenitor cells in the dentate gyrus, while removal of glucocorticoids by adrenalectomy (ADX) stimulates this process (Gould et al 1992, Cameron and Gould 1994). In our recent report (Wong and Herbert, 2004), we demonstrated that in addition to glucocorticoids’ effects on proliferation, raised levels reduced survival of newly-formed cells and a glucocorticoid-free environment promoted survival of these cells. However, whether glucocorticoids regulate differentiation of progenitor cells in the adult hippocampus has not been studied.

## Experimental procedures

### Animals

All procedures were carried out under Home Office (UK) license and guidelines. These stipulate the minimal use of animals and procedures required to minimize pain and suffering. Lister hooded male rats (Harlan, Bicester, UK), weighing around 250–300g at the beginning of the experiment, were used. Rats were housed three–four per cage on reversed 12-h light/dark cycles (lights off at 11:00 h). Ambient temperature was maintained at 21°±2°C. Food and water were available *ad libitum*. All surgeries were carried out under general anesthesia. ADX animals were given a choice of 0.9% saline and water to replenish salt loss.

### BrdU injection and corticosterone level manipulations

5-bromodeoxyuridine (BrdU) (Sigma), a thymidine analog incorporated into dividing cells during DNA synthetic phase (S-phase) of the cell cycle, was dissolved in 0.9% saline and injected i.p. (200mg/kg) at midday. A single BrdU injection paradigm was chosen to ensure that the population of cells labeled would come from the same time frame, and thus all newborn cells are in the same developmental phase at the start of the drug treatment.

To remove circulating glucocorticoids, bilateral ADX was performed under isoflurane anesthesia. Basal levels of corticosterone were replaced with s.c. implants of 1:1 corticosterone/cholesterol (Sigma) melted and molded into pellets weighing 200–220 mg. To increase plasma corticosterone above ‘basal’ levels, animals were given additional s.c. injections of corticosterone suspended in peanut oil at 40mg/kg/day, a dose shown to reduce neurogenesis. Reference plasma corticosterone levels of these different paradigms can be obtained in our recent publication (Wong and Herbert, 2005).

### Experimental procedures

#### Experiment 1

**Fig. 1:**
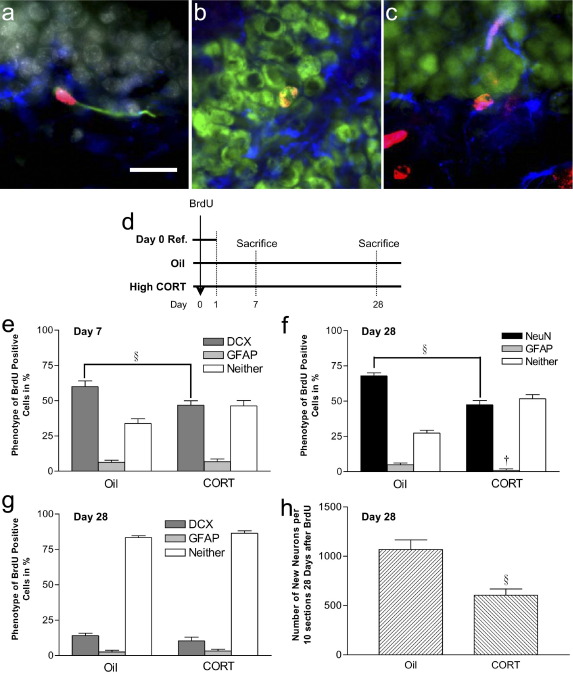
The morphology of (a) a BrdU/DCX double positive cell, (b) a BrdU/NeuN double positive cell, (c) a BrdU/GFAP double positive cell. (d) Design of experiment 1. (e) The percentage of phenotypes of BrdU-labeled cells at day 7. Corticosterone treatment significantly reduced the percentage of BrdU cells positive for the immature neuronal marker DCX at this time-point (^§^*F*_1,11_=6.40, *P*<0.03). (f) The phenotype of BrdU-labeled cells at day 28. Corticosterone treatment significantly reduced the percentage of BrdU cell positive for the mature neuronal marker NeuN at day 28 (^§^*F*_1,11_=31.9, *P*<0.0001). (g) The phenotype of BrdU-labeled cells at day 28. Corticosterone did not affect the proportion of BrdU cells expressing DCX at day 28. (h) The number of new neurons produced at day 28 calculated using the equation described in Experimental Procedures (data in Table 1). Corticosterone treatment significantly reduced the number of new neurons produced in the dentate gyrus 28 days after BrdU labeling (^§^*F*_1,11_=14.4, *P*<0.004).

Thirty animals, given a single injection of BrdU, were divided into three groups. Group 1 (*n*=6) was killed 24 h after the BrdU injection. Group 2 (*n*=12) received a daily injection of peanut oil, beginning 24 h after BrdU. Group 3 (*n*=12) received 40mg/kg/day corticosterone suspended in peanut oil. Animals (*n*=6) from each treatment group (2 and 3) were sampled at seven and 28 days after BrdU (Fig. 1d).

#### Experiment 2

**Fig. 2:**
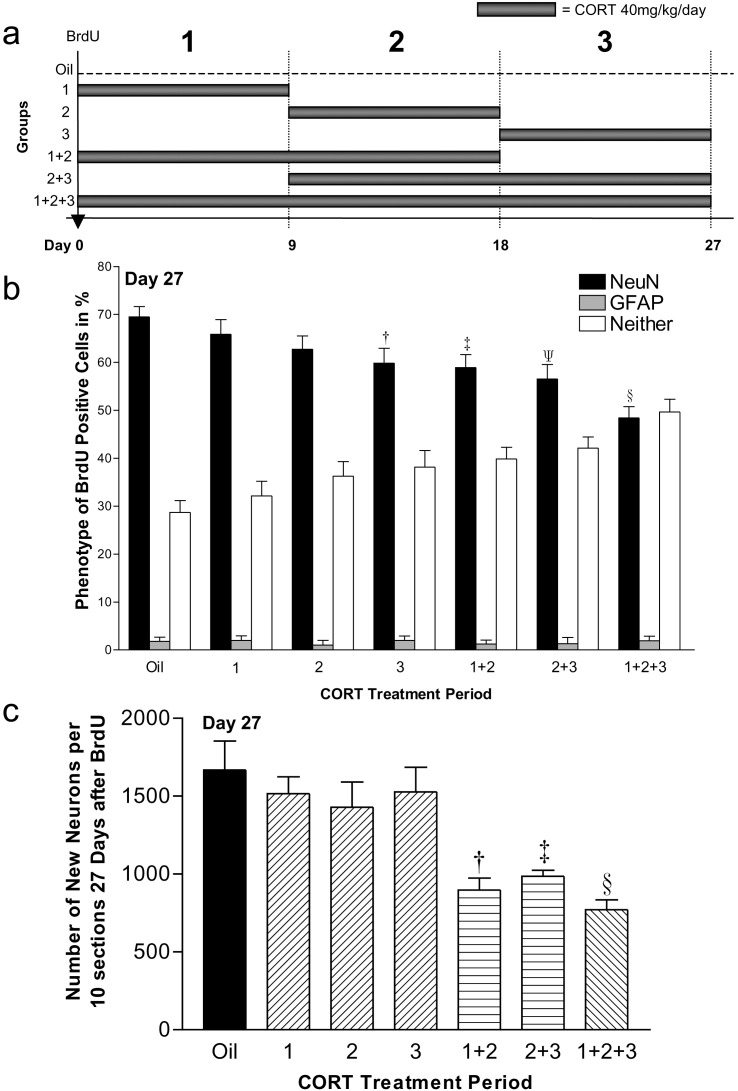
(a) Diagram depicting phased (9 days) corticosterone treatment for the seven groups over 27 days. (b) Corticosterone during period 3 significantly reduced neuronal differentiation (^†^*F*_1,15_=6.8, *P*<0.02), but not 1 or 2 alone. Giving corticosterone for 18 days or more significantly reduced proliferation (1+2+3: ^§^*F*_1,15_=44.3, *P*<0.0001; 1+2: ^‡^*F*_1,15_=9.62, *P*<0.008; 2+3: ^Ψ^*F*_1,15_=12.4, *P*<0.008). (c) Corticosterone treatment for 18 days or more (1+2 or 2+3 or 1+2+3) significantly reduced the numbers of new neurons 27 days after BrdU labeling (1+2: ^†^*P*<0.001; 2+3: ^‡^*P*<0.002; 1+2+3: ^§^*P*<0.0001).

Fifty-six intact rats were given BrdU as above, and then received various regimens of daily s.c. injections of either corticosterone (40mg/kg/day) or oil for 27 days. They were divided into seven groups of eight rats each. Group 1 received oil throughout. The next three groups received corticosterone for 9 days each, or oil otherwise. The start of corticosterone was staggered by nine days in each group (group two days 1–9, group three days 10–18, group four days 19–27). Group five received CORT on days 1–18, group six days 10–27, and group seven days 1–27. All rats were killed on day 28 (Fig. 2a).

#### Experiment 3

**Fig. 3:**
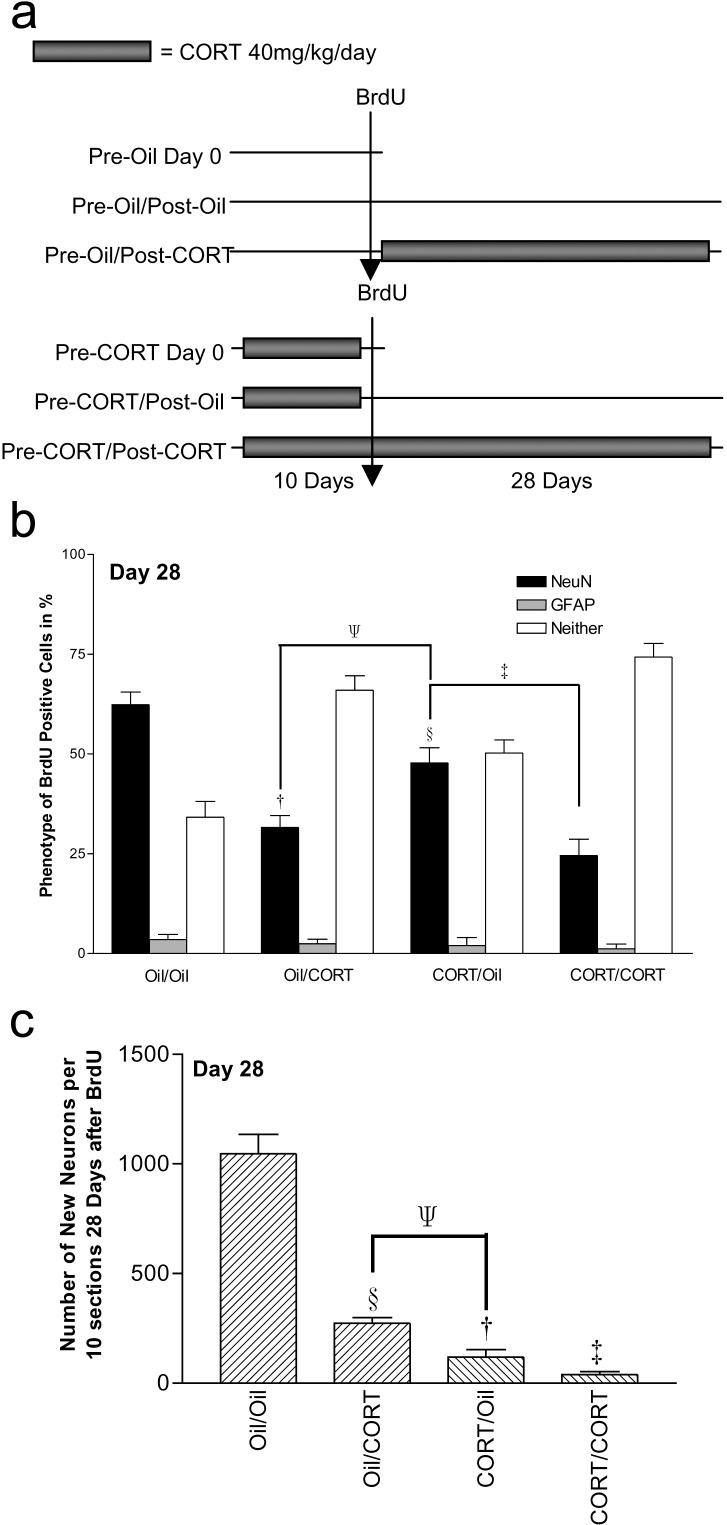
(a) Diagram showing the treatment paradigm for the six groups. (b) Both pre-mitotic and post-mitotic corticosterone significantly reduced neuronal differentiation (pre-mitotic: ^§^*P*<0.045; post-mitotic: ^†^*P*<0.0001; ^‡^*P*<0.001). (c) Both pre-mitotic and post-mitotic corticosterone treatments significantly reduced the number of new neurons formed 28 days after BrdU-labeling (^§^*P*<0.0001, ^†^*P*<0.0001, ^‡^*P*<0.0001). Treating animals with corticosterone before BrdU resulted in fewer new neurons than treating with corticosterone after BrdU (CORT/Oil vs Oil/CORT: ^§^*P*<0.021).

Rats were divided into two major groups (*n*=24 each). The first group was given daily oil injections for 10 days (pre-treatment), and then two injections of BrdU, 3 h apart, on day 10. This double injection protocol served to label a larger pool of proliferating cells to compensate for the suppressive effect of corticosterone on cell division. The second group (*n*=24) was given daily injections of corticosterone (40mg/kg/day) for 10 days, and BrdU as above. Each group was then divided into three sub-groups. Groups 1a and 2a were killed 24 h after the BrdU injection. Groups 1b and 2b were given another 28 days (post-treatment) of oil injections. Groups 1c and 2c were given 28 days of corticosterone (40mg/kg/day). The latter four sub-groups were perfused 24 h after the last injection (day 29) (Fig. 3a).

#### Experiment 4

**Fig. 4:**
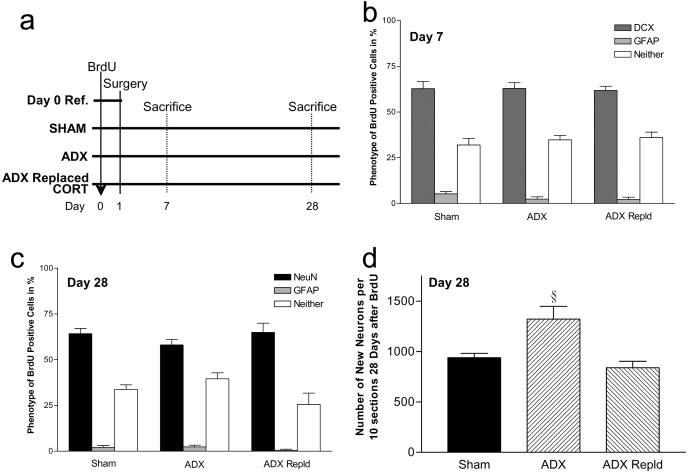
(a) Diagram showing the corticoid manipulations in the different groups of animals. (b, c) There was no observable effect by ADX on differentiation of the newly-formed cells as detected by DCX, NeuN and GFAP at day 7 and day 28. (d) Combining the survival data (Table 1) revealed that ADX significantly increased the number of new neurons formed in the dentate gyrus 28 days after BrdU labeling (^§^*P*<0.025).

Four groups of animals were used in this experiment (*n*=56). They all received a single injection of BrdU (200mg/kg, i.p.) to label the new-born cells. In group 1 (*n*=8), animals were killed 24 h after BrdU labeling, whereas in group 2 (*n*=16) they were sham-operated 24 h after BrdU and implanted s.c. with a cholesterol pellet. Eight rats were perfused at seven and 28 days after surgery. Group 3 (*n*=16) was bilaterally ADX 24 h after BrdU and implanted with a cholesterol pellet. Rats (*n*=8) were sampled at the same intervals. Group four (*n*=16) were also ADX, but received a 50% corticosterone pellet (200–250 mg). They were sampled at the same intervals (Fig. 4a).

#### Experiment 5

**Fig. 5:**
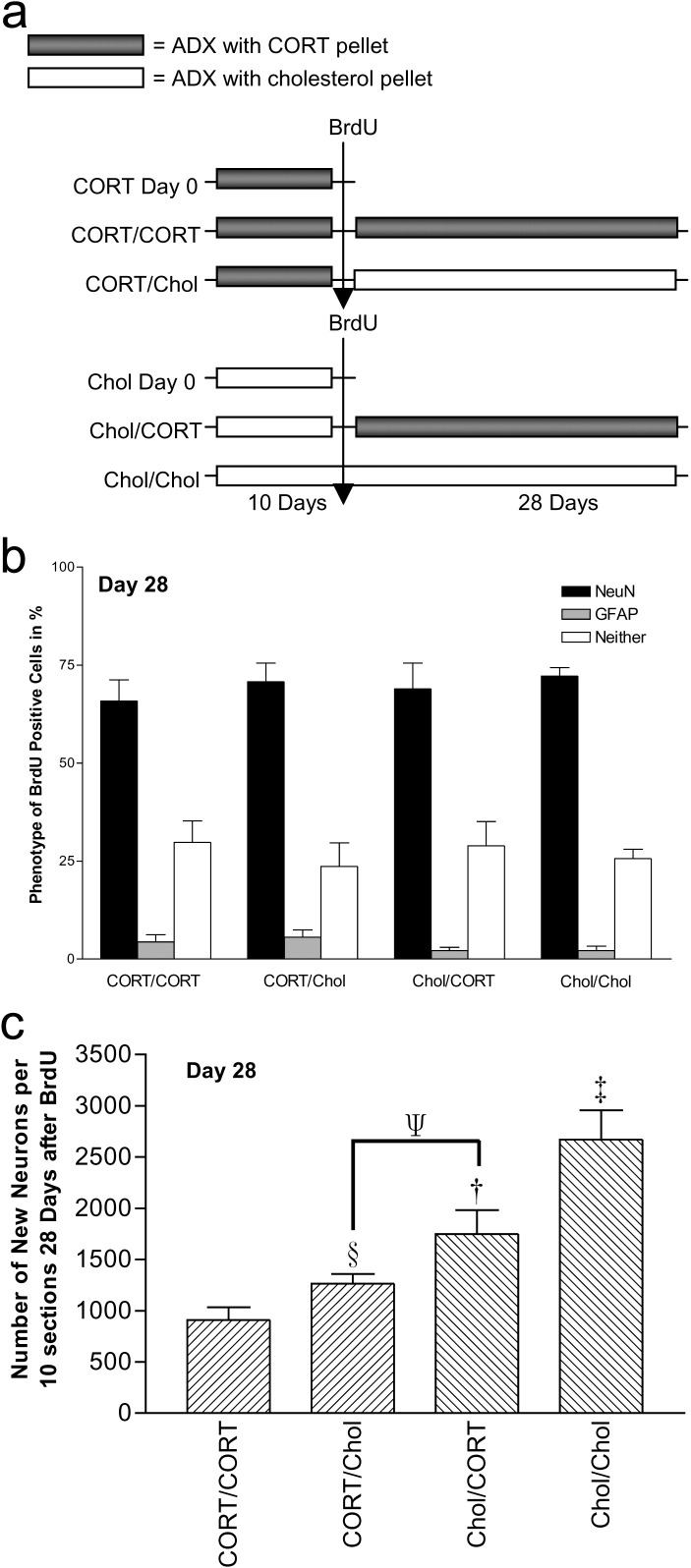
(a) Diagram depicting treatment paradigm for the six groups of rats. (b) Graph showing phenotype of BrdU cells at day 28. There was no difference in differentiation between the four day-28 treatment groups. (c) Combining the neuronal differentiation data with the survival data (Table 1) showed that ADX, whether before or after BrdU labeling, significantly enhanced the number of new neurons in the dentate gyrus (^§^*P*<0.034, ^†^*P*<0.009, ^‡^*P*<0.0001). ADX prior to BrdU administration gave a higher number of new neurons than ADX after BrdU administration 28 days later (^§^*P*<0.034).

All rats were adrenalectomized (ADX) (*n*=48), half were given basal corticosterone replacement (s.c. implant of 50% corticosterone pellet) and the others received a cholesterol pellet. Ten days after surgery, all animals received a single injection of BrdU. Each of the two original groups was divided into three sub-groups. The first two sub-groups (1a, 2a: *n*=8 per group) were killed 24 h after the BrdU injection. The second sub-groups (1b, 2b: *n*=8) had their corticosterone or cholesterol pellets removed and replaced with new, corticosterone pellet 24 h after BrdU. The third sub-groups (1c, 2c; *n*=8) had their corticosterone or cholesterol pellets removed and replaced with cholesterol pellets. Sub-groups b and c were perfused 29 days after BrdU (Fig. 5a).

### Fluorescence immunochemistry

Animals were given an overdose of pentobarbitone sodium and perfused transcardially with 0.1M PB followed by 4% paraformaldehyde in 0.01M KPBS (pH 7.4). Brains were removed, post-fixed for 4 h, and then immersed in 20% sucrose in KPBS overnight for cryoprotection against formation of water ice crystals during freezing. Coronal sections were cut using a freezing microtome through the entire dentate gyrus at 40μm and sections were stored in anti-freeze (1:1:2 glycerol:ethyleneglycol:0.1M PBS). All rinses were done in 0.01M KPBS. Sections were first incubated in 2 N HCl at 37°C, and incubated overnight at room temperature with rat anti-BrdU antibody (1:200, Accurate, USA) in 0.2% Triton-X (Sigma, UK) and 5% normal goat serum (Vector Laboratories, USA). The sections were rinsed, and incubated in Rodamine-RX conjugated anti-rat secondary antibodies (1:800; Jackson Laboratories, USA) for 60 min. Antibodies against doublecortin (DCX; marker for immature neuron; mouse; 1:500, Santa Cruz Biotechnology, USA), NeuN (marker for mature neuron; mouse; 1:800; Chemicon, USA) and GFAP (glial marker; rabbit; 1:200; Sigma) were applied in combination depending on the time-point examined and incubated overnight at room temperature. Alexa-488 conjugated anti-mouse (1:500; Molecular Probes, USA) or Alexa-647 conjugated anti-rabbit antibodies (1:500; Molecular Probes) were used in according to the primaries used. Sections were then mounted on to polylysine-coated slides and coverslipped with Vectamount (Vector Laboratories).

### Phenotype determination

All slides were randomized and coded prior to phenotype analysis. Analysis was performed using a Zeiss Axioskop 2 fluorescence microscope (Zeiss, Germany). A 10-layer Z-stack (0.762μm apart) with images from each color channel was captured with a 24-bit black/white digital camera using a 40× objective. Z-stacks were then deconvolved using Zeiss deconvolution software (a mathematical reconstruction model) to remove haze from adjacent sections. At least 30 BrdU cells from each brain were analyzed. Percentage neuronal differentiation was obtained from dividing the number of cells positive for neuronal marker by total number of cells analyzed.

### Estimation of total number of new Neu-N-stained neurons

The total number of new neurons in the sections examined was estimated using the following equation:

$$N_{{\text{E}}_{\text{st}}}=N_{0}\times {\%}_{\text{Sur}}\times {\%}_{\text{Diff}}$$

where *N*_Est_ represents the total number of new neurons; *N*_0_ refers to the number of cells labeled by BrdU 24 h after labeling; %_Sur_ is the number of surviving BrdU cells expressed as percentage of mean BrdU cell count 24 h after labeling presented in our previous report (Wong and Herbert, 2004) and summarized in Table 1; %_Diff_ represents the percentage of BrdU cells positive for the mature neuronal marker NeuN. The number of new neurons in the brain sections sampled for a particular animal was calculated using the corresponding survival and differentiation percentages.

### Statistical analysis

Data were analyzed by one, two or three-way ANOVA; all data presented as percentages were arcsine transformed before statistical analysis. Instances in which non-parametric tests were used are specified in the results section.

## Results

### Experiment 1: post-mitotic corticosterone reduced neuronal differentiation

Sections from animals killed on day-7 post-BrdU were triple-stained for BrdU, DCX and GFAP. A BrdU/DCX double positive cell is shown in Fig. 1a. Fig. 1e shows that corticosterone treatment for 7 days significantly reduced the percentage of BrdU cells immuno-positive for DCX at this time point (one-way ANOVA; *F*_1,11_=6.04, *P*<0.03). Corticosterone had no effect on differentiation into GFAP-stained cells (GFAP: *F*_1,11_=0.06, *P*=0.82). BrdU/NeuN and BrdU/GFAP cells are shown in Fig. 1b and c, respectively. Corticosterone also significantly reduced the percentage of BrdU cells immuno-positive for NeuN (*F*_1,11_=30.8, *P*<0.0001, Fig. 1f) and GFAP (*F*_1,11_=5.54, *P*<0.043) at day 28. To reveal whether this represented a delay in maturation, day 28 sections were triple-stained for BrdU/DCX/GFAP. There was no difference in percentage of BrdU-stained cells immuno-positive for DCX or GFAP or those staining for neither after corticosterone treatment (DCX: *F*_1,11_=1.43, *P*=0.26; GFAP: *F*_1,11_=0.14, *P*=0.72; neither: *F*_1,11_=2.15, *P*=0.18) (Fig. 1g). Combining the survival data in Table 1 and these data showed that the total number of new neurons was reduced by corticosterone treatment for 28 days (*F*_1,11_=14.4, *P*<0.004).

### Experiment 2: time-dependency of the effect of corticosterone on neuronal differentiation

Brain sections from all seven groups were triple stained for BrdU/NeuN/GFAP. Fig. 2b shows the phenotypes of the BrdU-positive cells by percentage for each group. Three-way ANOVA, with each period as the independent variable, showed that all three corticosterone treatment periods altered the phenotypes of the BrdU-stained cells (0–9: *F*_2,55_=5.51, *P*<0.023; 10–18: *F*_2,55_=5.74, *P*<0.021; 19–27: *F*_2,55_=15.9, *P*<0.0001), and that there was no interaction between the three treatment periods.

As expected from experiment 1, animals receiving 27 days of corticosterone had significantly lower percentage of NeuN positive BrdU-stained cells compared with animals receiving oil (one-way ANOVA, *F*_1,15_=43.6, *P*<0.0001). One-way ANOVA also revealed that corticosterone treatment during period 1 or 2 alone had no effect on the percentage of BrdU-stained cells expressing NeuN relative to the Oil group (1: *F*_1,15_=0.93, *P*=0.35; 2: *F*_1,15_=4.00, *P*=0.07). However, treatment during period 3 alone significantly reduced this percentage (*F*_1,15_=6.81, *P*<0.02). Although corticosterone treatment during period 1 or 2 had no significant effect on the phenotype of BrdU cells, when animals were treated with corticosterone during both periods 1 and 2, the percentage of BrdU/NeuN cells was significantly lowered (*F*_1,15_=9.43, *P*<0.009). This is also true of those receiving corticosterone during both periods 2 and 3 (*F*_1,15_=12.8, *P*<0.003). There was no detectable difference in GFAP phenotype (1: *F*_2,55_=0.24, *P*<0.88; 2: *F*_2,55_=0.34, *P*<0.57; 3: *F*_2,55_=0.021, *P*<0.89), and thus the proportion of BrdU-stained cells also immuno-reactive for NeuN correlates negatively with those that express neither NeuN nor GFAP.

The total numbers of new neurons being formed in the different treatment groups are shown in Fig. 2c. One-way ANOVA and post hoc comparisons of the seven groups showed that the none of the single 9-day corticosterone treatment had an effect (Bonferroni; 1: *P*=1.00; 2: *P*=1.00; 3: *P*=1.00), but both the 18-day and 27-day corticosterone treatments significantly reduced neuronal production (1+2: *P*<0.001; 2+3: *P*<0.002; 1+2+3: *P*<0.0001).

### Experiment 3: effect of high levels of corticosterone prior to progenitor proliferation on neuronal differentiation

In this experiment, animals received CORT either before or after BrdU labeling, or during both periods (see Fig. 3a). The percentages of each cell type for each group are shown in Fig. 3b. Corticosterone given both pre- and post-BrdU reduced NeuN phenotype. (Pre-treatment: *F*_1, 31_=9.73, *P*<0.005; *F*_1,31_=56.88, *P*<0.0001.) One-way ANOVA of the four groups demonstrated that corticosterone treatment significantly affected the percentage of BrdU-stained cells expressing NeuN (*F*_3,31_=24.8, *P*<0.0001) but did not alter the proportion of cells immuno-positive for GFAP (*F*_3,31_=0.52, *P*<0.67). Post hoc pair-wise comparisons showed that corticosterone pre-treatment reduced the percentage of BrdU/NeuN cells (Oil/Oil vs CORT/Oil; Bonferroni, *P*<0.041). Post-treatment with corticosterone irrespective of the pre-treatment significantly reduced the BrdU/NeuN percentage (Bonferroni, Oil/Oil vs Oil/CORT, ^†^*P*<0.0001; CORT/Oil Vs CORT/CORT, ^‡^*P*<0.002). There was no significant difference between the Oil/CORT and CORT/CORT groups (*P*=1.00). Importantly, the effect of post-mitotic corticosterone on neuronal differentiation was more pronounced than that of pre-mitotic treatment corticosterone (Oil/CORT vs CORT/Oil: *P*<0.040).

The total numbers of neurons generated from the four different paradigms are shown in Fig. 3c. Two-way ANOVA showed that corticosterone, both pre-treatment and post-treatment, significantly affected neuronal production (pre-treatment: *F*_3,31_=114.6, *P*<0.0001, post-treatment: *F*_3,31_=62.0, *P*<0.0001). There was an interaction between the two treatments (*F*_3,31_=40.9, *P*<0.0001). Post hoc analysis showed that animals receiving corticosterone treatment, whether before or after BrdU injection, had significantly fewer new neurons (Oil/CORT; *P*<0.0001; CORT/Oil: *P*<0.0001; CORT/CORT: *P*<0.0001). Treating animals with corticosterone before BrdU resulted in fewer new neurons than treatment with corticosterone after BrdU (CORT/Oil vs Oil/CORT: *P*<0.021).

### Experiment 4: effect of removal of endogenous glucocorticoids by ADX on neuronal differentiation

BrdU/DCX/GFAP-stained brain sections at day 7 from the three treatment groups were analyzed and the percentages of BrdU-stained cells immuno-positive for DCX, GFAP or neither are shown in Fig. 4b. One-way ANOVA showed that there was no difference between the three treatment groups in the percentages of BrdU-positive cells also positive for DCX, GFAP or neither (DCX: *F*_2,23_=0.053, *P*=0.95; GFAP: *F*_2,23_=2.00, *P*=0.16; Neither: *F*_2,23_=0.46, *P*=0.64). Sections from the day 28 survival groups were stained for BrdU/NeuN/GFAP. One-way ANOVA of the three treatment groups (Fig. 4c) showed no difference in the phenotypes of the BrdU-stained cells after treatment (NeuN: *F*_2,23_=1.21, *P*=0.32; GFAP: *F*_2,23_=0.98, *P*=0.40; Neither: *F*_2,23_=2.97, *P*=0.08). The number of new neurons formed in each group was estimated using both the survival and differentiation data, and are shown in Fig. 4d. One-way ANOVA and post hoc analysis showed that post-mitotic ADX significantly increased the total number of new neurons formed in the dentate gyrus (Bonferroni; *P*<0.025).

### Experiment 5: effect of a corticoid-free environment during proliferation

Phenotypic analysis of the four day 28 survival groups showed no difference in the phenotype of the BrdU-stained cells between the four treatments (Fig. 5b, one-way ANOVA, NeuN: *F*_3,31_=0.227, *P*=0.88; GFAP: *F*_3,31_=1.32, *P*=0.29; neither: *F*_3,31_=0.30, *P*=0.82). Estimating the total number of new neurons formed using the survival and differentiation data revealed an interesting trend (Fig. 5c); two-way ANOVA demonstrated that both pre-treatment and post-treatment were effective (Pre: *F*_3,23_=31.6, *P*<0.0001; Post: *F*_3,23_=10.2, *P*<0.003). There was no interaction between the two (*F*_3,23_=1.98, *P*=0.171). One-way ANOVA and post hoc analysis demonstrated that all non-corticosterone-replaced groups, either before or after BrdU, had higher numbers of new neurons 28 days after BrdU (CORT/Chol: ^§^*P*<0.034; Chol/CORT: ^†^*P*<0.009; Chol/Chol: ^‡^*P*<0.0001). A corticoid-free environment before BrdU labeling generated more neurons than a corticoid-free environment after BrdU labeling (Chol/CORT vs CORT/Chol: *P*<0.034).

## Discussion

Development of progenitors in the dentate gyrus can be divided into three distinct stages: proliferation, survival and differentiation. The effect of glucocorticoids, especially that of corticosterone, on the first two stages has been demonstrated previously (Gould et al 1992, Cameron and Gould 1994, Wong and Herbert 2004). In this report, we provide findings that fill a missing and crucial part of the developmental trajectory of progenitor cells residing in the dentate gyrus. Using fluorescence immunochemistry and cell-type specific markers, we show that corticosterone regulates the phenotype of the newly-formed cells in the dentate gyrus.

Experiment 1 showed that in addition to its depressive action on proliferation and survival, corticosterone given during the post-mitotic interval also reduces differentiation of the newly-formed cells into mature neurons. This could be demonstrated both at the day-7 and day-28 time points, at which time immature (DCX) and mature (NeuN) neuronal markers respectively are maximally expressed. The reduction in neuronal differentiation can be accounted for in two ways. Firstly, corticosterone may halt the differentiation process of certain populations of newly-formed cells, and thus the proportion (fate) of BrdU-positive cells also positive for these neuronal markers will be lower at any given time after BrdU labeling. Secondly, corticosterone may slow down the differentiation process, thereby introducing a ‘differentiation lag’ in this process. To clarify this, sections from the day-28 time point were triple-stained for the immature neuronal marker DCX because only a small proportion of newly-formed cells would be expected to express this marker at day-28 (Brown et al., 2003). If the observed reduction in neuronal differentiation after corticosterone was a consequence of a time window shift, one would expect to find a higher percentage of BrdU/DCX cells at the day-28 time point. However, our data suggested that this was not the case. Therefore, it is likely that corticosterone may convey a ‘stop’ signal to the differentiation process of the newly-formed cells, thus resulting in a decline in neuronal differentiation. Combining the survival data from our previous report (Wong and Herbert, 2004) with the differentiation data we present here, high levels of corticosterone present after progenitor division significantly lowered the number of new neurons formed in the samples we took of the dentate gyrus of the hippocampus. Since we have no data on rates of degeneration, it is also possible that increased apoptosis of maturing cells may contribute to the effects we describe here. A proportion of BrdU-labeled cells stained neither for GFAP, NeuN or DCX. The identity of these cells remains unknown, but the most likely explanation is that they are undifferentiated progenitor cells.

The regulation by corticosterone can be both direct (through receptors) and indirect, and candidates that mediate the effect of corticosterone include insulin-like growth factor-1 (IGF-1), brain-derived neurotrophin factor (BDNF), and epidermal growth factor (EGF), all of which have been shown to positively regulate adult neurogenesis and differentiation of progenitor cells (Kuhn et al 1997, Aberg et al 2000, Lee et al 2002). Glucocorticoids may alter neuronal differentiation via suppression of these pathways since glucocorticoids negatively modulate the expression of these signaling molecules and their receptors (Islam et al 1998, Schaaf et al 2000, Gubba et al 2004). The mechanism by which corticosterone regulates neuronal differentiation remains to be investigated.

There was an interesting difference between time-dependent actions of corticosterone on survival or differentiation of new cells. We showed previously that excess corticosterone given for 9 days during the first 18 days reduced survival, whereas treatment during the final 9 days did not. In contrast, this treatment regimen had no effect on the development of a neuronal phenotype, whereas corticosterone given during the final 9 days of the 28 day survival interval was effective. This suggests that there is a distinction between the corticoid-responsive processes regulating the two events, though the exact nature of either is still to be uncovered. Raised corticosterone, it seems, exerts maximal effect on survival during the early stages of progenitor development while its effect on differentiation is most powerful at a later stage. Combining the differentiation and survival data (Wong and Herbert, 2004) revealed that none of the 9-day treatment groups significantly reduced the total number of new neurons produced, but animals receiving corticosterone for 18 days or more resulted in significantly fewer neurons being produced.

High levels of corticosterone present prior to BrdU administration also reduced subsequent neuronal differentiation, as shown by experiment 3. Corticosterone treatment during the post-mitotic period in the experiment also reduced neuronal differentiation, confirming the results of the previous experiments. Interestingly, treating animals with corticosterone before progenitor proliferation also reduced the proportion of BrdU cells expressing the mature neuronal marker 28 days after labeling, irrespective of the treatment during the post-mitotic period. However, it is noteworthy that the suppressive effect of corticosterone on differentiation is stronger during the post-mitotic period. These actions are likely to be direct, since newly-formed cells express corticoid receptors at birth and the expression of these receptors increases over time (Garcia et al., 2004). However, any effect by glucocorticoids prior to progenitor division and during the early stages of development of these cells (when receptor expression is still low) are likely to be mediated by the surrounding local environment. Toward the day 28 time point, more differentiated cells may express corticoid receptors, so that the fact that post-mitotic corticosterone had a more prominent effect is possibly due to the increased sensitivity of more mature newly-formed cells to corticosterone from 19 to 27 days after birth. Nonetheless, estimation of total neuron production revealed that corticosterone treatment before progenitor division resulted in fewer neurons compared with animals receiving corticosterone during the post-mitotic period only. Combining the two treatments showed that neuron production in animals receiving corticosterone in both periods was merely 3.7% that of the control group.

Thus far, data in the literature and this report suggest that raised corticosterone is detrimental to all three stages of neurogenesis, proliferation, survival and differentiation, and that raised corticosterone reduces the number of neurons produced consequentially. Removal of glucocorticoids by ADX, on the other hand, positively regulates proliferation and survival of the progenitor cells (Wong and Herbert, 2004). However, triple staining in this report showed that ADX, and thus a relatively corticoid-free environment, did not favor increased proportional differentiation of newly-formed cells into neurons (experiments 4 and 5). Nonetheless, a corticoid-free environment during the post-mitotic period resulted in high number of neurons since overall survival is increased. Removal of glucocorticoids during both periods resulted in a 92.7% increase in the total number of new neurons in the sampled region relative to the control group. Although there is no evidence thus far to demonstrate that daily fluctuation of plasma corticoid levels would affect neurogenesis, it is important to note that high levels of glucocorticoids, as a result of physical or psychological stress, may have serious consequences on neurogenesis in the hippocampus. The dose of additional corticosterone we used (40mg/kg/day) is supra-physiological, and well into the ‘stress’ range. Ageing is also accompanied by raised circulating glucocorticoids (Sapolsky 1992, Lupien et al 1994) and it has been shown that hippocampal neurogenesis is reduced at old age, but data also exist to support manipulation of corticoid (by ADX) could reverse this suppression (Cameron and McKay, 1999).

A large body of literature suggests that excess corticosterone is detrimental to hippocampal functions such as working and spatial memories (Coburn-Litvak et al 2003, Woodson et al 2003). If new neurons in the adult hippocampus are indeed responsible for the formation of new memory (Shors et al., 2001), the reduction after corticosterone may account for the behavioral deficits observed in the learning models. Nonetheless, it is of immense interest to investigate whether a change in the number of new neurons in the dentate gyrus predicts a decline in hippocampal function, since new neurons numbers may be associated with synaptic changes and other molecular events such as LTP and LDP. Removal of glucocorticoids by ADX also impairs hippocampal-dependent learning (Armstrong et al 1993, Vaher et al 1994) and this is reversed by corticosterone replacement (Sousa et al., 1999). Moreover, ADX causes dramatic cell death in the granule cell layer (Cameron and Gould, 1996). Our observed increase in neuronal production in ADX animals may serve to compensate for this extensive loss. Whether the rate of ADX-induced cell loss is greater than the accompanying increase in new neuronal production, such that a net loss of granular neurons accounts for the decline in function, awaits further study.

The finding that glucocorticoids regulate neuronal differentiation in the dentate gyrus of the adult rat has important implications for those interested in the effects of stress on brain structure and function. This also provides further support for the recent suggestions that adult neurogenesis may be concerned with the psychopathology of major depressive disorder, a condition in which elevated glucocorticoids have been associated both as a risk factor for this condition, as well as one manifestation of the disease process itself (Goodyer et al 2000, Harris et al 2000, Duman 2004, Sapolsky 2004).

## Figures and Tables

**Table 1:** The number of new neurons in the dentate gyrus after the experimental treatments described in the text. This was estimated by **N_Ab_=N_0_×%_Sur_×%_Diff_** where **N_Ab_**=absolute number of new neurons, **N_0_**=number of cells labelled by BrdU 24 hours after labelling, **%_Sur_**=mean percentage surviving BrdU cells (data extracted from Wong and Herbert, 2004) and **%_Diff_**=percentage of BrdU immuno-positive NeuN.

Experiment	Group	*N*_0_	Mean %_Sur_	Mean %_Diff_	Total new neurons
1	Oil	2187.6±154	50.3±5.0	67.9±2.1	712.0±65.0
	High CORT	2187.6±154	34.4±3.8	47.3±3.1	403.5±42.6
2	Oil	2187.6±154	72.6±7.3	69.5±2.1	1111±125
	1	2187.6±154	58.3±10.3	65.9±3.0	1010±72.1
	2	2187.6±154	69.9±14.5	62.7±2.8	953±108
	3	2187.6±154	84.1±4.9	59.9±3.1	1019±105
	1+2	2187.6±154	46.0±2.0	58.9±2.7	598±51.3
	2+3	2187.6±154	53.8±2.3	56.6±3.0	657±26.1
	1+2+3	2187.6±154	48.6±3.4	48.4±2.3	513.9±42.7
3	Oil/Oil	3015±94	51.0±2.4	62.3±3.2	1047±87
	Oil/CORT	3012±94	29.1±1.2	31.6±3.0	273±27
	CORT/Oil	996±79	26.8±4.1	47.8±3.8	120±33
	CORT/CORT	996±79	11.5±2.5	24.5±4.1	39.3±13.7
4	Sham	2192±251	45.5±1.4	64.2±2.8	626±28.9
	ADX	2192±251	70.0±4.7	58.1±3.0	880±85.1
	ADX Repld	2192±251	41.2±2.8	64.9±5.0	560±42.1
5	CORT/CORT	2478±265	31.1±3.2	64.6±5.0	606±82.8
	CORT/Chol	2478±265	48.3±2.9	71.3±4.2	844±61
	Chol/CORT	7975±804	20.7±1.5	68.9±6.6	1167±155
	Chol/Chol	7975±804	31.1±3.5	72.2±2.2	1779±191

Note that the total number of new neurons presented in this table were calculated from taking the corresponding %_sur_ and %_diff_ from individual animals and substituting into the equation, and therefore these values may not necessarily be the same as those obtained by simply multiplying the mean percentages from each group.

## References

[bib1] Aberg M.A., Aberg N.D., Hedbacker H., Oscarsson J., Eriksson P.S. (2000). Peripheral infusion of IGF-I selectively induces neurogenesis in the adult rat hippocampus. J Neurosci.

[bib2] Altman J., Das G.D. (1966). Autoradiographic and histological studies of postnatal neurogenesis. I. A longitudinal investigation of the kinetics, migration and transformation of cells incorporating tritiated thymidine in neonate rats, with special reference to postnatal neurogenesis in some brain regions. J Comp Neurol.

[bib3] Armstrong J.N., McIntyre D.C., Neubort S., Sloviter R.S. (1993). Learning and memory after adrenalectomy-induced hippocampal dentate granule cell degeneration in the rat. Hippocampus.

[bib4] Brown J.P., Couillard-Despres S., Cooper-Kuhn C.M., Winkler J., Aigner L., Kuhn H.G. (2003). Transient expression of doublecortin during adult neurogenesis. J Comp Neurol.

[bib5] Cameron H.A., Gould E. (1994). Adult neurogenesis is regulated by adrenal steroids in the dentate gyrus. Neuroscience.

[bib6] Cameron H.A., Gould E. (1996). Distinct populations of cells in the adult dentate gyrus undergo mitosis or apoptosis in response to adrenalectomy. J Comp Neurol.

[bib7] Cameron H.A., McKay R.D. (1999). Restoring production of hippocampal neurons in old age. Nat Neurosci.

[bib8] Cameron H.A., Woolley C.S., McEwen B.S., Gould E. (1993). Differentiation of newly born neurons and glia in the dentate gyrus of the adult rat. Neuroscience.

[bib9] Coburn-Litvak P.S., Pothakos K., Tata D.A., McCloskey D.P., Anderson B.J. (2003). Chronic administration of corticosterone impairs spatial reference memory before spatial working memory in rats. Neurobiol Learn Mem.

[bib10] Corotto F.S., Henegar J.A., Maruniak J.A. (1993). Neurogenesis persists in the subependymal layer of the adult mouse brain. Neurosci Lett.

[bib11] de Quervain D.J., Roozendaal B., Nitsch R.M., McGaugh J.L., Hock C. (2000). Acute cortisone administration impairs retrieval of long-term declarative memory in humans. Nat Neurosci.

[bib12] Duman R.S. (2004). Depression: a case of neuronal life and death?. Biol Psychiatry.

[bib13] Eriksson P.S., Perfilieva E., Bjork-Eriksson T., Alborn A.M., Nordborg C., Peterson D.A., Gage F.H. (1998). Neurogenesis in the adult human hippocampus. Nat Med.

[bib14] Garcia A., Steiner B., Kronenberg G., Bick-Sander A., Kempermann G. (2004). Age-dependent expression of glucocorticoid- and mineralocorticoid receptors on neural precursor cell populations in the adult murine hippocampus. Aging Cell.

[bib15] Goodyer I.M., Herbert J., Tamplin A., Altham P.M. (2000). First-episode major depression in adolescents. Affective, cognitive and endocrine characteristics of risk status and predictors of onset. Br J Psychiatry.

[bib16] Gould E., Beylin A., Tanapat P., Reeves A., Shors T.J. (1999). Learning enhances adult neurogenesis in the hippocampal formation. Nat Neurosci.

[bib17] Gould E., Reeves A.J., Fallah M., Tanapat P., Gross C.G., Fuchs E. (1999). Hippocampal neurogenesis in adult Old World primates. Proc Natl Acad Sci USA.

[bib18] Gould E., Cameron H.A., Daniels D.C., Woolley C.S., McEwen B.S. (1992). Adrenal hormones suppress cell division in the adult rat dentate gyrus. J Neurosci.

[bib19] Gould E., McEwen B.S., Tanapat P., Galea L.A., Fuchs E. (1997). Neurogenesis in the dentate gyrus of the adult tree shrew is regulated by psychosocial stress and NMDA receptor activation. J Neurosci.

[bib20] Gubba E.M., Fawcett J.W., Herbert J. (2004). The effects of corticosterone and dehydroepiandrosterone on neurotrophic factor mRNA expression in primary hippocampal and astrocyte cultures. Brain Res Mol Brain Res.

[bib21] Harris T.O., Borsanyi S., Messari S., Stanford K., Cleary S.E., Shiers H.M., Brown G.W., Herbert J. (2000). Morning cortisol as a risk factor for subsequent major depressive disorder in adult women. Br J Psychiatry.

[bib22] Haughey N.J., Nath A., Chan S.L., Borchard A.C., Rao M.S., Mattson M.P. (2002). Disruption of neurogenesis by amyloid beta-peptide, and perturbed neural progenitor cell homeostasis, in models of Alzheimer’s disease. J Neurochem.

[bib23] Islam A., Ayer-LeLievre C., Heigenskold C., Bogdanovic N., Winblad B., Adem A. (1998). Changes in IGF-1 receptors in the hippocampus of adult rats after long-term adrenalectomy: receptor autoradiography and in situ hybridization histochemistry. Brain Res.

[bib24] Izquierdo I., Medina J.H. (1997). Memory formation: the sequence of biochemical events in the hippocampus and its connection to activity in other brain structures. Neurobiol Learn Mem.

[bib25] Kempermann G. (2002). Why new neurons? Possible functions for adult hippocampal neurogenesis. J Neurosci.

[bib26] Kempermann G., Kuhn H.G., Gage F.H. (1997). More hippocampal neurons in adult mice living in an enriched environment. Nature.

[bib27] Kuhn H.G., Dickinson-Anson H., Gage F.H. (1996). Neurogenesis in the dentate gyrus of the adult rat: age-related decrease of neuronal progenitor proliferation. J Neurosci.

[bib28] Kuhn H.G., Winkler J., Kempermann G., Thal L.J., Gage F.H. (1997). Epidermal growth factor and fibroblast growth factor-2 have different effects on neural progenitors in the adult rat brain. J Neurosci.

[bib29] Lee J., Duan W., Mattson M.P. (2002). Evidence that brain-derived neurotrophic factor is required for basal neurogenesis and mediates, in part, the enhancement of neurogenesis by dietary restriction in the hippocampus of adult mice. J Neurochem.

[bib30] Liu J., Solway K., Messing R.O., Sharp F.R. (1998). Increased neurogenesis in the dentate gyrus after transient global ischemia in gerbils. J Neurosci.

[bib31] Lupien S., Lecours A.R., Lussier I., Schwartz G., Nair N.P., Meaney M.J. (1994). Basal cortisol levels and cognitive deficits in human aging. J Neurosci.

[bib32] Malberg J.E., Schechter L.E. (2005). Increasing hippocampal neurogenesis: a novel mechanism for antidepressant drugs. Curr Pharm Des.

[bib33] Monje M.L., Mizumatsu S., Fike J.R., Palmer T.D. (2002). Irradiation induces neural precursor-cell dysfunction. Nat Med.

[bib34] Nadel L., Moscovitch M. (1997). Memory consolidation, retrograde amnesia and the hippocampal complex. Curr Opin Neurobiol.

[bib35] Nixon K., Crews F.T. (2002). Binge ethanol exposure decreases neurogenesis in adult rat hippocampus. J Neurochem.

[bib36] Peissner W., Kocher M., Treuer H., Gillardon F. (1999). Ionizing radiation-induced apoptosis of proliferating stem cells in the dentate gyrus of the adult rat hippocampus. Brain Res Mol Brain Res.

[bib37] Pham K., Nacher J., Hof P.R., McEwen B.S. (2003). Repeated restraint stress suppresses neurogenesis and induces biphasic PSA-NCAM expression in the adult rat dentate gyrus. Eur J Neurosci.

[bib38] Santarelli L., Saxe M., Gross C., Surget A., Battaglia F., Dulawa S., Weisstaub N., Lee J., Duman R., Arancio O., Belzung C., Hen R. (2003). Requirement of hippocampal neurogenesis for the behavioral effects of antidepressants. Science.

[bib39] Sapolsky R.M. (1992). Do glucocorticoid concentrations rise with age in the rat?. Neurobiol Aging.

[bib40] Sapolsky R.M. (2004). Is impaired neurogenesis relevant to the affective symptoms of depression?. Biol Psychiatry.

[bib42] Schaaf M.J., De Kloet E.R., Vreugdenhil E. (2000). Corticosterone effects on BDNF expression in the hippocampus. Implications for memory formation. Stress.

[bib43] Shors T.J., Miesegaes G., Beylin A., Zhao M., Rydel T., Gould E. (2001). Neurogenesis in the adult is involved in the formation of trace memories. Nature.

[bib44] Sousa N., Madeira M.D., Paula-Barbosa M.M. (1999). Corticosterone replacement restores normal morphological features to the hippocampal dendrites, axons and synapses of adrenalectomized rats. J Neurocytol.

[bib45] Vaher P., Luine V., Gould E., McEwen B.S. (1994). Adrenalectomy impairs spatial memory in rats. Ann N Y Acad Sci.

[bib46] Wong E.Y.H., Herbert J. (2004). Glucocorticoids environment: a determining factor for neural progenitors survival in the adult hippocampus. Eur J Neurosci.

[bib47] Wong E.Y.H., Herbert J. (2005). Roles of mineralocorticoid and glucocorticoid receptors in the regulation of progenitor proliferation in the adult hippocampus. Eur J Neurosci.

[bib48] Woodson J.C., Macintosh D., Fleshner M., Diamond D.M. (2003). Emotion-induced amnesia in rats: working memory-specific impairment, corticosterone-memory correlation, and fear versus arousal effects on memory. Learn Mem.

